# Scarlet Fever in Newport, R. I.

**Published:** 1879-05

**Authors:** 


SCARLET FEVER AT NEWPORT, R. I.—In view of the prevalence of scarlet fever at Newport, R. I., Dr. H. R. Storer has call the attention of the board of aldermen to their neglect of sanitary precaution in that town. He says the board has refused to transfer its powers to a competent board of health; children of infected families are allowed to attend public and private schools; open funerals are permitted, and alleged defects in the construction of the sewers are not corrected.—*Boston Med. & Surg. Jour.*

